# The Quaternary evolutionary history, potential distribution dynamics, and conservation implications for a Qinghai–Tibet Plateau endemic herbaceous perennial, *Anisodus tanguticus* (Solanaceae)

**DOI:** 10.1002/ece3.2019

**Published:** 2016-02-24

**Authors:** Dong‐Shi Wan, Jian‐Ju Feng, De‐Chun Jiang, Kang‐Shan Mao, Yuan‐Wen Duan, Georg Miehe, Lars Opgenoorth

**Affiliations:** ^1^ State Key Laboratory of Grassland Agro‐EcosystemSchool of Life SciencesLanzhou UniversityLanzhou730000GansuChina; ^2^ College of Plant SciencesXinjiang Production & Construction Corps Key Laboratory of Protection and Utilization of Biological Resources in Tarim BasinTarimu UniversityAlarXinjiangChina; ^3^ Key Laboratory for Bio‐resources and Eco‐environment of Ministry of EducationCollege of Life ScienceSichuan UniversityChengdu610064China; ^4^ Key Laboratory of Biodiversity and BiogeographyKunming Institute of BotanyChinese Academy of SciencesLanhei Road 132, HeilongtanKunming650204YunnanChina; ^5^ Faculty of Biology and GeologyUniversity of Marburg35032MarburgGermany

**Keywords:** Glacial refugia, nuclear internal transcribed spacer, phylogeographic pattern, plastid DNA, species distribution modeling

## Abstract

Various hypotheses have been proposed about the Quaternary evolutionary history of plant species on the Qinghai–Tibet Plateau (QTP), yet only a handful of studies have considered both population genetics and ecological niche context. In this study, we proposed and compared climate refugia hypotheses based on the phylogeographic pattern of *Anisodus tanguticus* (three plastid DNA fragments and nuclear internal transcribed spacer regions from 32 populations) and present and past species distribution models (SDMs). We detected six plastid haplotypes in two well‐differentiated lineages. Although all haplotypes could be found in its western (sampling) area, only haplotypes from one lineage occurred in its eastern area. Meanwhile, most genetic variations existed between populations (*F*_ST_ = 0.822). The SDMs during the last glacial maximum and last interglacial periods showed range fragmentation in the western area and significant range contraction in the eastern area, respectively, in comparison with current potential distribution. This species may have undergone intraspecific divergence during the early Quaternary, which may have been caused by survival in different refugia during the earliest known glacial in the QTP, rather than geological isolation due to orogenesis events. Subsequently, climate oscillations during the Quaternary resulted in a dynamic distribution range for this species as well as the distribution pattern of its plastid haplotypes and nuclear genotypes. The interglacial periods may have had a greater effect on *A. tanguticus* than the glacial periods. Most importantly, neither genetic data nor SDM alone can fully reveal the climate refugia history of this species. We also discuss the conservation implications for this important Tibetan folk medicine plant in light of these findings and SDMs under future climate models. Together, our results underline the necessity to combine phylogeographic and SDM approaches in future investigations of the Quaternary evolutionary history of species in topographically complex areas, such as the QTP.

## Introduction

During the Quaternary climatic oscillations, plant species in the temperate region of the Northern Hemisphere have undergone repeated range contractions and expansions to track favorable habitats (e.g., Hewitt 2000, 2004; Stewart et al. 2010; Gavin et al. 2014). Fossil records and phylogeographic surveys of temperate plant species in Europe and North America, especially tree and shrub species, have suggested a typical scenario of southward retreats during glacial periods followed by rapid range expansion northwards during inter‐/postglacial periods. Although recent research has revealed a more complex pattern, with additional cryptic refugia or microrefugia at higher latitudes, no doubt remains concerning the existence of important southern glacial refugia and prominent routes of interglacial range expansions or recolonizations northwards (e.g., Stewart et al. 2010; Tzedakis et al. 2013; de Lafontaine et al. 2014). In contrast, the variations in Quaternary phylogeographic histories of plant species in topographically complex regions, such as the Qinghai–Tibet Plateau (QTP), are much more pronounced (e.g., Zhang et al. 2005; Opgenoorth et al. 2010; Qiu et al. 2011).

As the highest and one of the most extensive plateaus on earth, the QTP extends for ca. 2.5 million km^2^ with an average altitude above 4000 m (a.s.l.), and the Quaternary climatic oscillations probably affected species on the QTP more than those in other regions of similar latitude (Qiu et al. 2011; Liu et al. 2012). Geological evidence suggests that at least four Quaternary glaciations occurred on the QTP (Shi et al. 1995, 1997, 1998, 2000; Zheng et al. 2002): The Xixibangma Glacial, which occurred around 1.6 million years ago (Mya) and overlapped with the Euburonian glacial stage in northern Europe, is the earliest recognizable glaciation on the plateau (Zheng et al. 2002); the largest glaciation on the QTP (the Naynayxungla Glaciation) started around 1.2 Mya and reached its maximum between 0.8 and 0.6 Mya (Shi and Ren 1990; Zhou and Li 1998; Zheng et al. 2002). After that, several cycles of climatic oscillations, which resulted in mountainous glaciations only, might have continued to the Holocene (Shi et al. 1998). Meanwhile, it has been proposed that an abrupt and rapid uplift of the QTP occurred ca. 3.4 Mya around the middle of the Pliocene (Li et al. 1979, 1995, 1996; Cui et al. 1996; Shi et al. 1998, 1999), which may have also acted as a driving force in shaping the current population genetic structure and distribution range of species in this area (e.g., Liu et al. 2014; Wen et al. 2014). This relatively recent orogenesis event as well as extensive QTP uplifts during the Miocene resulted in a network of high mountains and deep valleys, leading to species diversification (e.g., Liu et al. 2002, 2006; Wang et al. 2009a; Sun et al. 2012; Wen et al. 2014) and intraspecific divergence (e.g., Wang et al. 2009b; Jia et al. 2011; Yang et al. 2012) in this area.

To date, two major types of hypotheses have been developed and tested on the Quaternary histories of plant species on the QTP. In an “in situ survival” hypothesis, cold‐resistant species survived through glacial periods in multiple refugia or microrefugia on the plateau (e.g., Wang et al. 2009b, 2014; Opgenoorth et al. 2010; Ma et al. 2014); in a “*tabula rasa*” hypothesis, species retreated to eastern or southeastern declivities during glacial periods and recolonized onto the plateau during interglacial or postglacial periods (e.g., Zhang et al. 2005; Meng et al. 2007; Yang et al. 2008; Wu et al. 2010). However, up until now, only a limited portion of species on the QTP have been investigated phylogeographically (Qiu et al. 2011; Liu et al. 2012, 2014), and further investigations are required for a comprehensive understanding of the Quaternary histories of this area's flora.

Meanwhile, previous studies suggested that species possessing different attributes (e.g., with respect to cold‐tolerance, drought‐tolerance, life cycle, and dispersal ability) may have experienced different Quaternary (late Neogene) histories and they may also exhibit different responses to future climate change (e.g., Shafer et al. 2010; Stewart et al. 2010; Alvarado‐Serrano and Knowles 2014). This emphasizes the need to consider ecological niche context when developing and testing hypotheses relating to species' evolutionary histories during Quaternary climate oscillations, as well as when proposing conservation and management strategies for them. Species distribution modeling (SDM, alternatively known as ecological niche modeling), which produces spatial predictions of a species' historical and current range using the coordinates of collection localities and Geographic Information System (GIS) maps of environmental data such as temperature and precipitation, provides a means for generating practical phylogeographic hypotheses (Richards et al. 2007; Elith and Leathwick 2009; Hickerson et al. 2010; Alvarado‐Serrano and Knowles 2014). SDM and phylogeographic analyses are complementary as the inferences from one approach can be explored and potentially validated by the other (Richards et al. 2007). SDMs provide solutions for an inherent weakness of phylogeographic analyses, namely that candidate refugia outside the present species distribution range can rarely be identified. This is especially helpful when fossil records are absent or ambiguous (Schorr et al. 2012). To date, SDM approaches have been employed to address the phylogeographic histories and future distribution dynamics of biota in various regions, including Europe (e.g., Alsos et al. 2009; Schorr et al. 2012; Bystriakova et al. 2014), North America (e.g., Carstens and Richards 2007; Rojas‐Soto et al. 2012), South America (Fontanella et al. 2012; Gallardo et al. 2013), Asia (e.g., Qi et al. 2012; Poudel et al. 2014), and Australasia (e.g., Buckley et al. 2009; Marske et al. 2009; Costion et al. 2015). However, only a handful of studies have employed both phylogeographic and SDM approaches to elucidate the Quaternary history of high‐altitude herbaceous species on the QTP (e.g., Liang et al. 2015; Wang et al. 2015; see also two recent studies on tree species: Li et al. 2013; Sun et al. 2015).

*Anisodus tanguticus* (Maxim.) Pasher (Solanaceae) is a perennial octoploid (Tu et al. 2005) distributed on the QTP at altitudes ranging from 2800 to 4200 m (He et al. 2004). This species and its congeners are an important group of traditional medicinal plants in China that produce anticholinergic alkaloids such as hyoscyamine and scopolamine (e.g., Yang 1991; Zhang and Wang 2002). Although this plant is of medical importance and is subject to ongoing loss of habitat (Yang 1991; Zheng et al. 2008), the geographic distribution of its genetic variation is poorly studied. To date, the only work addressing its population genetic structure was performed with RAPDs and suggested that ten sampled populations were clustered into two groups (Zheng et al. 2008). Meanwhile, recent molecular dating of the *Anisodus* genus has suggested that *A. tanguticus* diverged from the most recent common ancestor (MRCA) of the other three *Anisodus* species ca. 4.35 Mya (95% HPD: 1.70–7.80 Mya; Tu et al. 2010). Thus, the evolutionary history of this high‐mountain herb at the population level merits closer investigation.

In this study, we employed three maternally inherited plastid DNA regions and the biparentally inherited nuclear internal transcribed spacer (ITS) region (Alvarez and Wendel 2003; Clarkson et al. 2004), to survey genetic variation among 354 specimens from 32 populations sampled throughout almost the entire geographic distribution of *A. tanguticus*. We also employed SDMs to reconstruct the potential distribution of this species during the present day, the last glacial maximum (LGM), the last interglacial (LIG), and the future (2050 and 2070). We aimed (1) to survey the phylogeographic pattern of *A. tanguticus*, (2) to elucidate its Quaternary evolutionary history based on two lines of evidence, genetic data and SDMs, and (3) to discuss conservation implications for this folk medicine plant in light of phylogeographic pattern and future distribution dynamics.

## Materials and Methods

### Ethics statement

Although *A. tanguticus* is listed as a Grade II Nationally Protected Plant Species in China, all leaf samples employed in this study were collected from public areas, where no permission for collection of tiny numbers of leaves for DNA extraction is needed.

### Population sampling

**Figure 1:**
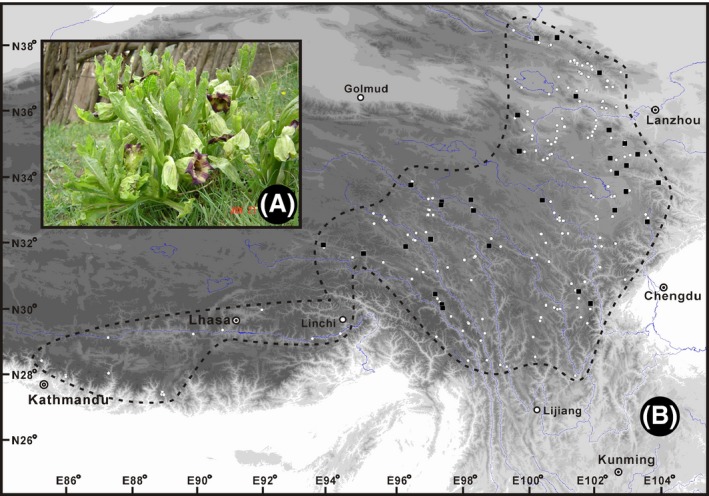
A photograph of *Anisodus tanguticus* plant (A), presumed distribution range of the species and sampling location of the 32 populations in this study (B). Black dashed lines mark the presumed distribution range of this species according to herbarium records, and black squares represent sampled populations; main cities that fell within the scope are also shown. Our sampling covered the main part of the potential distribution range of this species, see also Fig. 4A.

According to the Flora of China (Zhang et al. 1994), *A. tanguticus* mainly occurs on sunny grassy slopes in the eastern and southeastern parts of the QTP in China. In this study, we sampled 32 populations that represent its major distribution range (Fig. 1). Note, however, that the natural distribution of this species to the west of Lhasa merits closer investigation as herbarium samples from this area are fragmentary and rare (our SDMs also suggested that our sampling covered most of the potential geographic distribution of this species, see “Results” for details).

A summary of provenance and voucher specimen information for all populations is given in Table 1, and their geographic origins are shown in Fig. 1. Leaves from 12 individuals per population were collected from most sampling locations, while three to 11 individuals per population were sampled for five locations (Table 1). Sampled individuals were at least 50 m away from each other. Fresh leaves of each individual were collected, dried immediately in silica gel, and then stored at room temperature. The latitude, longitude, and altitude of each population were measured using an Etrex GIS (Garmin, Taiwan, China). Voucher specimens were deposited at the Herbarium of the Northwest Plateau Institute of Biology, the Chinese Academy of Sciences, Xining, China.

**Table 1:** Locations of studied populations of *Anisodus tanguticus*, number of individuals sampled, number of each plastid haplotype, and estimates of gene diversity (*H*_E_) per population. Note that two populations were used in the genetic survey but not the SDMs (too close to another sampling population), and five populations were used in the SDMs but not the genetic survey

No.	Voucher	SDMs	Location	Latitude	Longitude	Altitude	*N*	*H*_E_	Haplotypes					
									A	B	C	D	E	F
1	LiuJQ‐0842	Yes	Chenduo, Qinghai	N. 33°19′	E. 98°15′	3660 m	12	0.8485	2	2	2	0	4	2
2	LiuJQ‐0854	No	Yushu, Qinghai	N. 33°10′	E. 97°22′	3870 m	11	0.6364	1	5	0	0	5	0
3	LiuJQ‐0967	Yes	Qumalai, Qinghai	N. 33°46′	E. 96°26′	3930 m	12	0.5455	0	6	0	0	6	0
4	AO‐094	Yes	Yushu, Qinghai	N. 33°09′	E. 97°21′	3960 m	10	0.6889	2	3	0	0	5	0
5	LiuJQ‐0876	Yes	Yushu, Qinghai	N. 32°07′	E. 97°02′	3540 m	12	0.0000	0	0	0	0	12	0
6	LiuJQ‐1021	Yes	Nangqian, Qinghai	N. 31°54′	E. 96°17′	2900 m	12	0.1667	0	0	0	1	11	0
7	LiuJQ‐1058	Yes	Dingqing, Xizang	N. 31°41′	E. 95°00′	3990 m	10	0.0000	0	0	0	0	10	0
8	LiuJQ‐1081	Yes	Suoxian, Xizang	N. 31°57′	E. 93°47′	4100 m	12	0.5303	0	0	0	5	7	0
9	LiuJQ‐1230	Yes	Bangda, Xizang	N. 30°27′	E. 97°10′	4370 m	8	0.0000	0	0	0	8	0	0
10	LiuJQ‐06300	No	Zuogong, Xizang	N. 30°08′	E. 97° 23′	4070 m	12	0.0000	0	0	0	12	0	0
11	LiuJQ‐2671	Yes	Bangda, Xizang	N. 30°06′	E. 97°24′	4070 m	12	0.0000	0	0	0	12	0	0
12	AO‐115	Yes	Dege, Sichuan	N. 31°55′	E. 98°48′	3670 m	12	0.0000	0	0	0	12	0	0
13	AO‐110	Yes	Shiqu, Sichuan	N. 32°59′	E. 98°20′	3970 m	12	0.0000	12	0	0	0	0	0
14	LiuJQ‐05103	Yes	Daofu, Sichuan	N. 30°32′	E. 101°31′	3580 m	3	0.0000	3	0	0	0	0	0
15	LiuJQ‐05099	Yes	Kangding, Sichuan	N. 30°10′	E. 101°52′	3750 m	12	0.0000	12	0	0	0	0	0
16	LiuJQ‐05116	Yes	Songpan, Sichuan	N. 32°39′	E. 103°36′	2860 m	12	0.0000	12	0	0	0	0	0
17	LiuJQ‐06029	Yes	Hongyuan, Sichuan	N. 33°00′	E. 102°36′	3470 m	12	0.0000	12	0	0	0	0	0
18	LiuJQ‐1804	Yes	Dari, Qinghai	N. 33°18′	E. 100°25′	4020 m	12	0.0000	12	0	0	0	0	0
19	LiuJQ‐1870	Yes	Ruoergai, Sichuan	N. 33°34′	E. 102°57′	3450 m	12	0.0000	12	0	0	0	0	0
20	LiuJQ‐05117	Yes	Ruoergai, Sichuan	N. 33°50′	E. 103°56′	3460 m	12	0.0000	12	0	0	0	0	0
21	LiuJQ‐05122	Yes	Ruoergai, Sichuan	N. 34°07′	E. 102°40′	3130 m	12	0.0000	12	0	0	0	0	0
22	LiuJQ‐05128	Yes	Zhuoni, Gansu	N. 34°21′	E. 102°58′	3260 m	12	0.0000	12	0	0	0	0	0
23	LiuJQ‐05133	Yes	Lintan, Gansu	N. 34°42′	E. 103°18′	2850 m	12	0.3030	10	2	0	0	0	0
24	LiuJQ‐05135	Yes	Hezuo, Gansu	N. 35°01′	E. 102°55′	2950 m	12	0.0000	12	0	0	0	0	0
25	AO‐061	Yes	Guashize, Qinghai	N. 35°26′	E. 102°26′	3150 m	12	0.3030	2	10	0	0	0	0
26	LiuJQ‐06036	Yes	Luqu, Gansu	N. 34°35′	E. 102°28′	3120 m	7	0.0000	0	7	0	0	0	0
27	LiuJQ‐1734	Yes	Maqin, Qinghai	N. 34°47′	E. 99°43′	3660 m	12	0.0000	0	12	0	0	0	0
28	AO‐086	Yes	Xinghai, Qinghai	N. 35°53′	E. 99°40′	3760 m	12	0.0000	12	0	0	0	0	0
29	AO‐074	Yes	Huangzhong, Qinghai	N. 36°27′	E. 101°26′	2900 m	5	0.0000	5	0	0	0	0	0
30	LiuJQ‐04011	Yes	Menyuan, Qinghai	N. 37°10′	E. 102°09′	3100 m	12	0.0000	12	0	0	0	0	0
31	LiuJQ‐B01	Yes	Qilian, Qinghai	N. 38°14′	E. 100°52′	3500 m	12	0.0000	12	0	0	0	0	0
32	LiuJQ‐0715	Yes	Qilian, Qinghai	N. 38°13′	E. 100°15′	3200 m	12	0.0000	12	0	0	0	0	0
33	SDM only	Yes	Kangding, Sichuan	N30.31314	E101.56142	3794 m	–	–	–	–	–	–	–	–
34	SDM only	Yes	Dege, Sichuan	N31.91736	E098.60975	3375 m	–	–	–	–	–	–	–	–
35	SDM only	Yes	Litang, Sichuan	N30.22150	E099.82353	4209 m	–	–	–	–	–	–	–	–
36	SDM only	Yes	Jiangdang, Xizang	N31.33743	E098.13356	3822 m	–	–	–	–	–	–	–	–
37	SDM only	Yes	Mangkang, Xizang	N29.73816	E098.72279	3540 m	–	–	–	–	–	–	–	–
Total							354		193	47	2	50	60	2

Alt., altitude; *N*, number of individuals analyzed.

### DNA extraction, amplification, sequencing, and ITS‐RFLP

Total DNA was extracted from the silica gel‐dried leaves using the modified 2× CTAB procedure (Doyle and Doyle 1987). Three plastid regions, *rpl*20*‐rps*12, *psb*A*‐trn*H and *trn*L*‐*F, and the nuclear ITS region were amplified with universal primers (White et al. 1990; Taberlet et al. 1991; Hamilton 1999; Shaw et al. 2005). Polymerase chain reaction (PCR) was performed in a 25‐*μ*L volume, containing 10–40 ng plant DNA, 50 mm Tris–HCl, 1.5 mm MgCl_2_, 250 μg/mL BSA, 0.5 mm dNTPs, 2 *μ* m of each primer, and 0.75 units of Taq polymerase (Takara, Dalian, China). The annealing temperature set for each pair of primers ranged from 53 to 56 °C. PCR was performed with initial denaturing of 7 min at 95 °C followed by 36 cycles of 1 min at 94 °C, 1 min of annealing, 1 min of elongation at 72 °C, and ending with an 8 min extension at 72 °C. PCR purification kits provided by CAS Array (Shanghai, China) were used to purify the PCR products. Sequencing reactions and successive purifications were performed, and capillary analyses were run on an ABI 3130XL (Lanzhou University, Lanzhou, China) following the manufacturer's protocols.

Direct sequencing of the ITS region was carried out for all specimens. Among these, successful sequencing results were obtained for 78 specimens only, most likely due to the wide occurrence of hybrid ITS sequences and the octoploid nature of the species. In these 78 sequences, we found ITS sequences that belong to two significantly different lineages, which differ from each other in a 9‐bp insertion (CCGCGGCGC), and more than 10 mutations that are strongly correlated with the presence or absence of the insertion. We therefore performed an RFLP analysis to discriminate between these two lineages for all populations. SacII was employed to cut the restriction site “CCGC|GG.” The restriction enzyme digestions were performed for 1 h at 37 °C in a 10 *μ*L reaction system, containing 1 *μ*L SacII, 2 *μ*L 10 × T, 2 *μ*L 10% BSA, and 5 *μ*L PCR products. The digestion products were subjected to 1.5% agarose gel electrophoresis, and a DL2000 DNA ladder marker (Takara) was adopted to determine the size of the DNA fragments. The size of resulting fragments for all individuals was carefully checked so as to eliminate false positives.

The sequences produced were gathered and aligned using Clustal X version 1.83 (Thompson et al. 1997), followed by manual adjustments in Mega 5 (Tamura et al. 2011) according to the similarity criterion (Simmons 2004). A matrix of combined plastid sequences was constructed for all individuals examined, and plastid haplotypes were identified in DnaSP version 5 (Librado and Rozas 2009). All DNA sequences newly generated in this study have been deposited in the GenBank database under Accession Numbers KM208637–KM208656.

### Phylogenetic analyses

Phylogenetic analyses of combined plastid sequences were performed by maximum parsimony (MP) using PAUP* 4.0b10 (Swofford 2002). MP analyses (equally weighted characters and nucleotide transformations) involved a heuristic search strategy with 100 replicates of random addition sequences, in combination with ACCTRAN character optimization, MULPARS + TBR branch swapping and STEEPEST DESCENT options on. Bootstrap values (Felsenstein 1985) were calculated from 1000 replicates using a single heuristic search with simple addition with TBR and MULPARS options on in PAUP*. The relationships among detected plastid haplotypes were analyzed using TCS 1.21 (Clement et al. 2000). This program constructs haplotype networks by implementing the statistical parsimony algorithm described by Templeton et al. (1992). TCS was run with the default parsimony connection limit of 95%.

### Dating divergence between lineages

We used the plastid dataset to estimate divergence times. We first performed a likelihood ratio test of the hypothesis of equal substitution rates in the plastid dataset in MEGA 5 (Tamura et al. 2011), and a molecular clock hypothesis was not rejected at a significance level of 0.05 (*P* = 0.61). BEAST version 1.6.1 (Drummond and Rambaut 2007) was used to estimate divergence time among lineages, employing a Bayesian MCMC chain. Under the GTR model of nucleotide substitution with a gamma distribution and four rate categories, the “Coalescent: Constant Size” tree prior model was implemented with a “Strict Clock” molecular clock model. For all analyses, posterior distributions of parameters were approximated using two independent MCMC analyses of 500,000,000 generations with 20% burn‐in. The program tracer 1.5.0 (Rambaut and Drummond 2009) was used to check effective sample size, and the program TreeAnnotator 1.6.1 (part of the BEAST 1.6.1 package) was used to combine all samples and converge and/or summarize the output results. Finally, a tree with ages for each node and their 95% credible intervals (i.e., 95% highest posterior density intervals in the BEAST manual) was displayed and modified in FigTree 1.3.1 (Rambaut 2009). As there is no paleobotanical information for *Anisodus* or related genera that could be used to calibrate tree nodes, we calculated the substitution rate of *Anisodus* species based on the plastid dataset and the divergence timescale of Tu et al. (2010). Accordingly, a substitution rate of 1.64 × 10^−9^ substitutions/site/year (95% HPD: 4.05 × 10^−10^–3.29 × 10^−9^) was applied to the age estimation. This substitution rate falls into the range 1.00 × 10^−9^–3.00 × 10^−9^ substitutions/site/year for an average synonymous substitution rate of plastid regions in plants (Wolfe et al. 1987; Gonzales et al. 2008), this is in agreement with the fact that *Anisodus* are herbaceous perennials.

### Analysis of population structure

For the ptDNA data, estimates of unbiased genetic diversity (*H*_E_) equivalent to expected heterozygosity for diploid data (Weir 1996) were calculated for each population based on haplotype composition using the methods of Nei (1987). The parameters of population diversity (*H*_S_, *H*_T_) and differentiation (*G*_ST_, *N*_ST_) were estimated following the methods described by Pons and Petit (1996), using the program PERMUT (http://www.pierroton.inra.fr/genetics/labo/Software/Permut). Two differentiation parameters (*G*_ST_, *N*_ST_) were compared to infer the occurrence of significant phylogeographic structure using a permutation test with 1000 permutations. Genetic structure was further examined by analysis of molecular variance (AMOVA) (Excoffier et al. 1992) as implemented in ARLEQUIN version 3.0 (Excoffier et al. 2005), and genetic differentiation among haplotypes was considered by adding a distance matrix. SAMOVA analysis, which considered both plastid haplotype relationships and geographic distance, was simulated for *K* = 2 to *K* = 8. Three population subdivision schemes were employed in the AMOVA analyses of both datasets. In addition, Monmonier's maximum‐difference algorithm in BARRIER v2.2 (Manni et al. 2004) was used to identify biogeographic boundaries or areas exhibiting the largest genetic discontinuities between population pairs. The robustness of these boundaries was assessed by running BARRIER on 100 replicates of population average pairwise difference matrices. These matrices were generated by bootstrapping of haplotype sequences in SEQBOOT (Felsenstein 2005) and subsequent analyses in ARLEQUIN version 3.0 (Excoffier et al. 2005).

### Species distribution modeling

To reconstruct the past and current distribution ranges of *A. tanguticus*, we employed the SDM approach to determine potential distributions in the LIG (ca. 140–120 Ka before present), the LGM (ca. 21–18 Ka before present), the present day, and in the future (2050 and 2070 A.D.) using the maximum entropy approach as implemented in the program MAXENT 3.3.3k (Phillips et al. 2006). The species occurrence data with geographic coordinates for *A. tanguticus* at present are the same as in Table 1. In total, 35 points were used in these modeling analyses (field survey dataset). We also compiled species occurrence data from the Chinese Virtual Herbarium (www.cvh.org.cn). After eliminating potentially misidentified samples, decreasing sampling density in easier‐access areas, and including the above 35 points, a total of 157 points were used in subsequent modeling (herbarium samples plus field survey dataset). The altitude variable, and all bioclimatic layers with 19 bioclimatic variables at a resolution of 2.5 arc‐min during the LGM (MIROC: Hasumi and Emori 2004; CCSM: Collins et al. 2006), at present and in the future (2050, rcp45, average over 2041–2060; 2070, rcp85, average over 2061–2080) (CMIP5: Meehl et al. 2009), and LIG bioclimatic layers at a resolution of 30 arc‐second (Otto‐Bliesner et al. 2006) were obtained from the WorldClim database (available at http://www.worldclim.org/download; Hijmans et al. 2005). The LIG bioclimatic layers were resampled to achieve the same resolution using DIVA‐GIS 7.5 (http://www.diva-gis.org/). To avoid strong collinearity of environmental variables, which could lead to model over‐fitting, Pearson correlation for bioclimatic variables was conducted and the less correlated variables were retained (Pearson correlation value, <0.8). In total, altitude and eight bioclimatic variables (Mean Diurnal Range, Isothermality, Mean Temperature of Warmest Quarter, Temperature Annual Range, Precipitation of Wettest Quarter, Precipitation of Driest Quarter, Precipitation Seasonality, and Precipitation of Warmest Quarter) were used to model the distribution of *A. tanguticus*.

Species distribution models were constructed according to current environmental factors and then projected for the three different time periods. We employed 20 replicates using 80% of the distribution coordinates for training and 20% for testing, and the model with the best AUC value was adopted. A jackknife test was performed to measure the percent contributions of bioclimatic variables to the prediction for the distributional models. At the same time, the “10 percentile presence” threshold approach was employed because presence‐only data were available. Graphics for each predicted SDM were drawn using DIVA–GIS 7.5.

## Results

### Plastid DNA sequence variation

**Figure 2:**
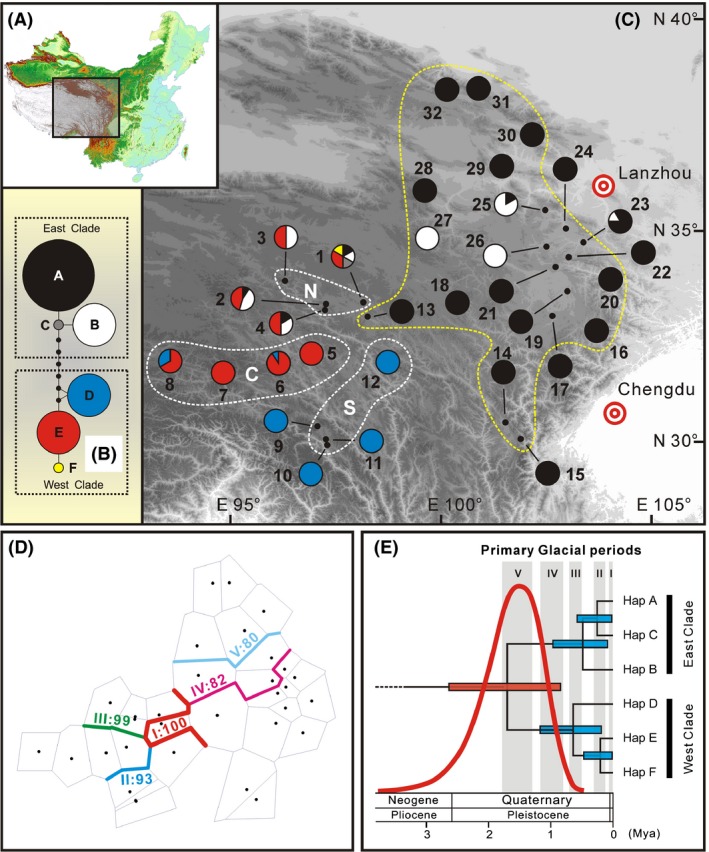
Map of the sampling areas, the phylogenetic network, and geographic distribution, genetic barriers, and divergence timescale of plastid haplotypes in *Anisodus tanguticus* (Maxim.) Pasher. (A) Sampling area (*shaded*). (B) Phylogenetic network of the six plastid haplotypes examined. (C) Sampling site and plastid haplotype frequencies in surveyed populations of *A. tanguticus*. (D) Genetic barriers to plastid haplotypes between different sampling areas. (E) Divergence timescale among all plastid haplotypes. In (C), white dashed lines represent the western sampling area (populations 1–12), which comprised three parts: the northwestern sampling area (N; populations 1–4), the central‐western sampling area (C; populations 5–8), and the southwestern sampling area (S; populations 9–12); yellow dashed lines delineate the eastern sampling area (populations 13–32). In (E), vertical gray zones represent four known glacial periods in the Qinghai–Tibet Plateau.

Among the 354 individuals, the total alignment length of the three plastid DNA sequences examined was 1660 bp. Nucleotide substitutions occurred at 10 sites, but no indel existed (Table 2). In combination, these polymorphisms identified a total of six plastid haplotypes. While 24 populations were fixed for a single haplotype, the remaining eight were polymorphic (Table 1, Fig. 2C). Only two haplotypes (A and B) were present among populations located in the eastern part of the species' geographic distribution (eastern group: populations 13–32), while all the haplotypes were found in western group (populations 1–12) (Fig. 2C). In the eastern populations, haplotype A was fixed in 16 populations and also occurred in two other populations; this frequent haplotype is absent from only two populations. However, two major haplotypes, D and E, were also found in the western group; these two haplotypes occurred in six and eight populations in this group, respectively. Two rare haplotypes, C and F, were recorded only in Pop 1 (Fig. 2C).

**Table 2:** Variable sites among the six plastid haplotypes of *Anisodus tanguticus* according to the aligned sequences of three plastid DNA fragments. Sequences are numbered from the 5′ to the 3′ end in each region

Haplotypes	Nucleotide position									
	*psb*A‐*trn*H					*trn*L‐F				*rps*12
	1	2	2	3	3	0	6	7	7	1
	7	0	9	0	6	2	5	2	8	8
	0	4	3	1	8	2	7	6	2	1
A	G	A	C	G	A	C	C	C	C	A
B	T	T	A	T	C	T	T	C	T	G
C	G	A	C	G	A	C	C	T	C	A
D	G	T	C	G	C	T	T	C	T	G
E	G	A	C	G	A	C	C	C	C	G
F	T	T	A	T	C	T	T	C	T	A

Defining *Przewalskia tangutica* Maxim. and *Atropa belladonna* Linn. as out‐groups, the most parsimonious tree was reconstructed (results not shown) based on 1620 constant, 31 uninformative, and nine informative sites (length = 43; CI = 0.95; RI = 0.87). Two well‐supported clades were apparent in the MP tree, and these were fairly well supported by bootstrap values (91% and 83%). One clade contained haplotypes A, B, and C with a bootstrap value of 91%; another clade contained haplotypes D, E, F, and the bootstrap value was 83%. Except for the infrequent haplotypes C and F, haplotypes comprising these two clades were geographically (mainly) distributed in the eastern and western groups of *A. tanguticus*, respectively (Fig. 2B and C). In the haplotype network, the two major clades were separated by at least five substitutions (Fig. 2B). Therefore, we referred to these two lineages of the plastid dataset as the East Clade (haplotypes A, B, C) and the West Clade (haplotypes D, E, F).

### RFLP analysis of ITS sequences

**Figure 3:**
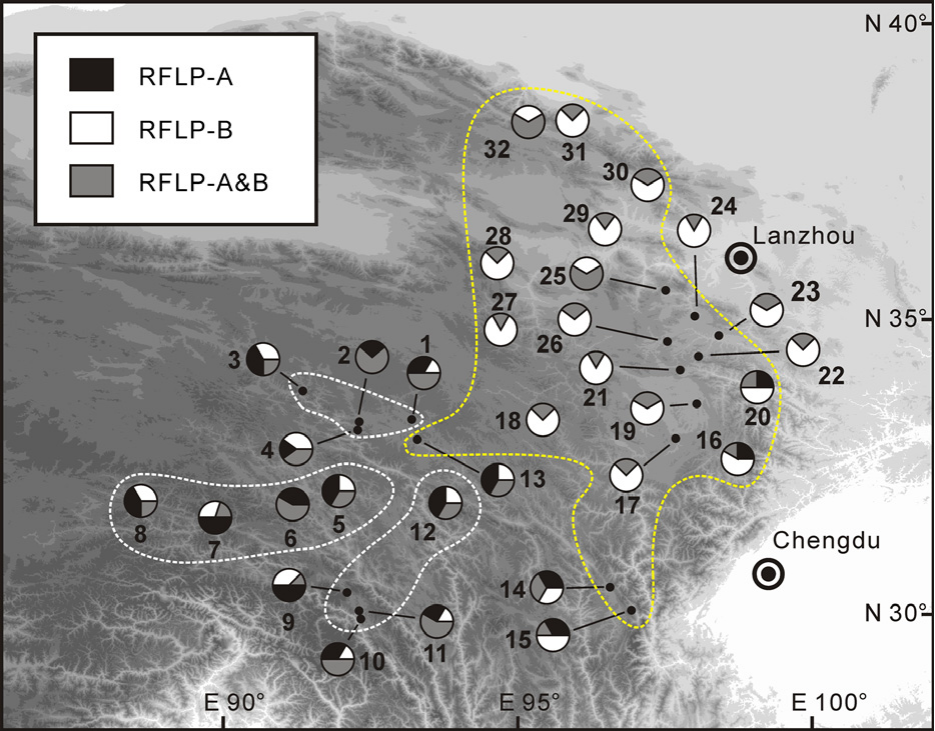
The geographic distribution of nuclear ITS‐RFLP types observed in *Anisodus tanguticus*. In each pie chart that represents a population, black represents individuals that possess RFLP‐A, white represents individuals that possess RFLP‐B, and gray represents individuals that possess both alleles. The division of sampling areas follows that of plastid haplotypes as shown in Fig. 2.

According to the DNA sequences of 78 specimens (see Materials and Methods section), the RFLP analysis succeeded in discerning two ITS lineages by producing a single band for one allele (RFLP‐A, no indel), two bands for another allele (RFLP‐B, with indel), and three bands for heterozygotes (RFLP‐A and B, i.e., additive). As shown in Fig. 3, RFLP‐A was found in most populations, and RFLP‐B was detected in the western populations and southern fringe of the eastern populations. However, individuals with both alleles were found in all populations.

### Age estimates for divergence of haplotypes

Two parallel runs of BEAST age estimations based on plastid sequences resulted in highly convergent results, the effective sampling size (ESS) for each parameter of each run was well above 200, and the ESS of node ages are all above 1 × 10^5^. The final set of age estimates were derived from a combination of the two parallel runs.

According to BEAST age estimations based on the plastid dataset and a substitution rate of 1.64 × 10^−9^ substitutions/site/year (note that age estimates below were derived by secondary calibration and should be treated with caution), the West and the East Clades diverged from each other 1.69 Mya (95% HPD: 0.83–2.62 Mya). Within the West Clade, the divergence time between haplotype D and the MRCA of haplotypes E and F was estimated to be 0.63 Mya (95% HPD: 0.18–1.16 Mya), and the latter pair diverged from each other 0.19 Mya (95% HPD: 0–0.46 Mya). Within the East Clade, the divergence time between haplotype B and the MRCA of haplotypes A and C was estimated to be 0.47 Mya (95% HPD: 0.08–0.95 Mya), and the latter pair diverged from each other 0.24 Mya (95% 95% HPD: 0.01–0.56 Mya). When applying a substitution rate of 1.00 × 10^−9^ and 3.00 × 10^−9^ substitutions/site/year, the divergence between the two clades was estimated to have occurred earlier (2.84 Mya, 95% HPD: 1.29–4.60 Mya) or latter (0.95 Mya, 95% HPD: 0.43–1.54 Mya), respectively.

### Population structure analyses

Plastid haplotype diversity (*H*_E_) was estimated based on haplotype frequencies in each population (Table 1). Population differentiation within the sampling range was very high (*N*_ST_ = 0.823 ± 0.0656) and was higher than *G*_ST_ (0.802 ± 0.0584; 0.01 < *P *<* *0.05), indicating a phylogeographic structure across the entire sampling range. Total genetic diversity *H*_T_ (0.661 ± 0.0643) across all populations was much higher than average within‐population diversity *H*_S_ (0.131 ± 0.0426), and consequently, population differentiation across the sampling range of the species was high.

Analyses based on the plastid dataset revealed that genetic differentiation among populations across the whole distribution of the species was much higher than that within populations (*F*_ST_ = 0.822) (Table 3). The grouping pattern of hierarchical AMOVA was consistent with relationships of plastid haplotypes and their geographic distribution (Table 3; Fig. 2). When *K* = 2, SAMOVA assigned populations 13–32 (eastern group) and populations 1–12 (western group) to independent groups, and the proportion of variation among groups was 75.38% (Table 3). When *K* = 3, SAMOVA assigned populations 1–8, populations 9–12 and the eastern populations (13–32) to independent groups, and the proportion of variation among groups was 79.45% (Table 3). When *K* = 4, SAMOVA assigned populations 1–4 (northwestern populations), populations 5–8 (central‐western populations), populations 9–12 (southwestern populations), and the eastern populations (13–32) to independent groups, and the proportion of variation among groups was 83.44% (Table 3; Fig. 2C). When *K* ≥ 5, SAMOVA generated a negative value for *F*_SC_ and we therefore did not consider these population subdivision schemes. Because *K* = 4 partitioned the highest proportion of variation among groups, we adopted this as the best population subdivision scheme (Fig. 2C).

**Table 3:** Analyses of molecular variance (AMOVA) for plastid haplotypes in *Anisodus tanguticus*

Source of variation	df	SS	VC	PV (%)	Fixation index
(i) all populations					
Between populations	31	541.55	1.5	82.18	*F*_ST_ = 0.822^a^
Within populations	322	108.26	0.34	17.82	
Total	353	649.81	1.89	100.00	
(ii) western (P 1–12) vs. eastern (P 13–32)					
Between groups	1	419.88	2.49	75.38	*F*_ST_ = 0.887^a^ *F*_SC_ = 0.542^a^ *F*_CT_ = 0.754^a^
Between populations within groups	30	156.17	0.44	13.33	
Within populations	320	119.32	0.37	11.29	
Total	351	695.37	3.30	100.00	
(iii) northwestern and central‐western (P 1–8) vs. southwestern (P 9–12) vs. eastern (P 13–32)					
Between groups	2	478.75	2.49	79.45	*F*_ST_ = 0.881^a^ *F*_SC_ = 0.422^a^ *F*_CT_ = 0.795^a^
Between populations within groups	29	97.30	0.27	8.67	
Within populations	320	119.32	0.37	11.88	
Total	351	695.37	3.14	100.00	
(iv) northwestern (P 1–4) vs. central‐western (P 5–8) vs. southwestern (P 9–12) vs. eastern (P 13–32)					
Between groups	3	523.29	2.57	83.44	*F*_ST_ = 0.879^a^ *F*_SC_ = 0.270^a^ *F*_CT_ = 0.834^a^
Between populations within groups	28	52.76	0.14	4.48	
Within populations	320	119.32	0.37	12.08	
Total	351	695.37	3.09	100.00	

df, degrees of freedom; SS, sum of squares; VC, variance components; *F*_*ST*_, correlation within populations relative to total; *F*_CT_, correlation of plastid haplotypes within groups relative to total; *F*_SC_, correlation within populations relative to groups.

a *P *<* *0.01.

Strong genetic barriers were detected among the eastern (Fig. 2D), northwestern, central‐western, and southwestern sampling areas, and these three barriers received high bootstrap values (93–100%). Although two genetic barriers were detected within the eastern sampling area (Fig. 2D), their support values were relatively low (80–82%).

### Species distribution models for *A. tanguticus*

**Figure 4:**
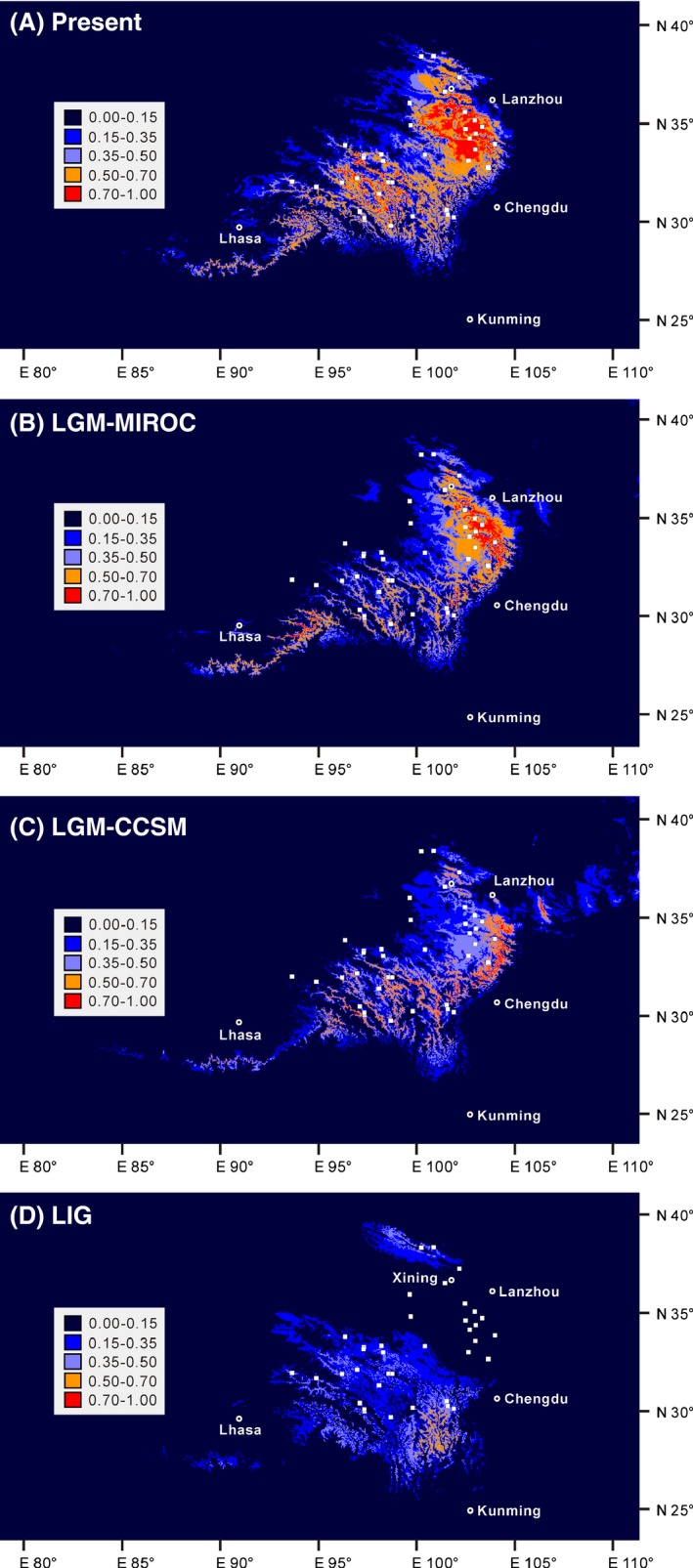
Distribution dynamics of *Anisodus tanguticus* during the present day, the LGM, and the LIG based on species distribution modeling using MAXENT. Predicted distributions are shown for (A) the present‐day model, (B) the LGM‐MIROC model, (C) the LGM‐CCSM model, and (D) the LIG model. White squares represent presence locations (field survey data), and white circles (with black dot in the center) indicate the main cities. LGM, last glacial maximum; LIG, last interglacial.

MAXENT models based on the field survey dataset revealed that the projected present‐day distribution is consistent with our collection records. According to the average over the 20 replicates of the MAXENT runs, the areas under the ROC curve (AUC) for *A. tanguticus* have values of 0.984 ± 0.006, 0.978 ± 0.005, 0.983 ± 0.006, 0.980 ± 0.003, 0.982 ± 0.006, and 0.979 ± 0.006 for the present‐day model, the LGM‐MIROC model, the LGM‐CCSM model, the LIG model, the 2050 (rcp45), and the 2070 (rcp85) models, respectively, indicating that our models differed greatly from random expectation. Variable jackknife analyses suggested that Altitude (53.2%), Precipitation of Warmest Quarter (25.3%), Mean Diurnal Range (8.7%), and Mean Temperature of Warmest Quarter (6.0%) are the environmental variables that contributed most to potential distribution modeling. Both LGM models (MIRCO and CCSM) generated potential distributions (habitat suitability index > 0.15) that are largely consistent with each other (Fig. 4B and C), but LGM‐CCSM generated a narrower predicted distribution with high habitat suitability (habitat suitability index > 0.5).

Based on the field survey dataset, the predicted distributions of the species during the present day, LGM and LIG periods are illustrated in Fig. 4. Compared to the present‐day model, both LGM models for the species showed significant contraction and fragmentation in the western sampling area. When considering predicted distribution with high habitat suitability (>0.5), the eastward range contraction was predicted in both the LGM‐MIROC and LGM‐CCSM models, but the latter was much more significant than the former. In contrast, the LIG distribution of *A. tanguticus* showed a significant southward contraction and predicted habitat suitability (>0.5) distributions are located in a narrow area close to the southeastern edge of the QTP (Fig. 4D).

**Figure 5:**
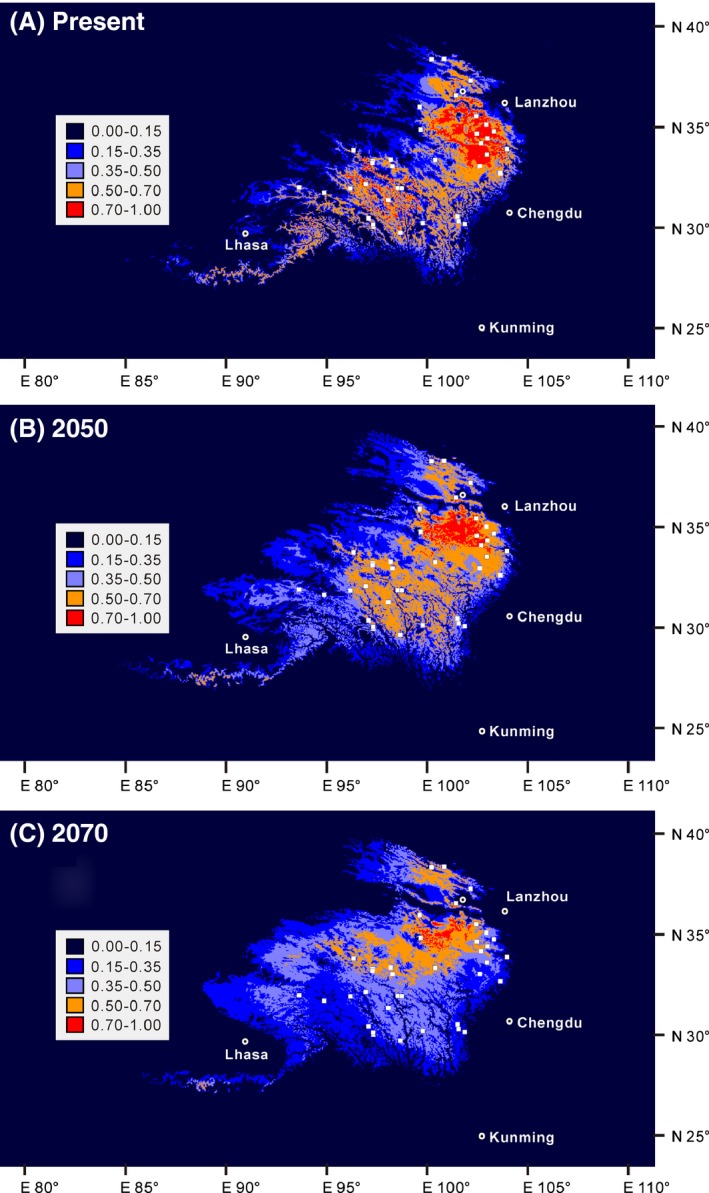
Potential distribution of *Anisodus tanguticus* under future global warming scenarios. Predicted distributions are shown for (A) the present‐day model, (B) the 2050 (rcp45) model, and (C) the 2070 (rcp85) model. White squares indicate presence locations (field survey data), and white circles (with black dot in the center) indicate the main cities.

Meanwhile, the potential distribution of *A. tanguticus* at present and under future climate change scenarios (2050, 2070) was also modeled based on the field survey dataset (Fig. 5). When considering predicted distribution based on high habitat suitability (>0.5), the 2050 model (rcp45, which assumes a radiative forcing value of 4.5/Wm^2^) predicted a more or less similar distribution, but the 2070 model (rcp85, which assumes a radiative forcing value of 8.5/Wm^2^) predicted a significant northward contraction compared to the present‐day model. Note that more than 26 populations that were sampled in this study fell out of the high habitat suitability (>0.5) predictions in the 2070 model, including all populations from the central‐western and southwestern sampling area.

It is noteworthy that the MAXENT models based on herbarium samples plus the field survey dataset (Figs S1, S2) generate similar potential distributions compared to those based on the field survey dataset alone (Figs. 4, 5).

## Discussion

### Phylogeographic pattern

Our genetic survey of 352 individuals across 32 populations of the QTP endemic perennial species, *A. tanguticus*, detected six plastid haplotypes. Phylogenetic analyses suggested that these six haplotypes were clustered into two well‐supported clades by phylogenetic analyses (West Clade, East Clade; Fig. 2B and C). The distribution of haplotypes showed a significant phylogeographic structure (*N*_ST_ > *G*_ST_, 0.01 < *P *<* *0.05). SAMOVA suggested that the sampled populations could be divided into four groups, and that distribution of haplotype diversity is uneven among them (Fig. 2C). First, five of six plastid haplotypes were found in the northwestern sampling area (populations 1–4). Furthermore, among the remaining three groups, a single haplotype was detected in the southwestern sampling area (populations 9–12), and up to two haplotypes from the West and East Clades were detected in the central‐western (populations 5–8) and eastern (populations 13–32) sampling area, respectively. Note that in the eastern sampling area, 18 of 20 populations only possess a single haplotype. Finally, two haplotypes from the West Clade were found in the southwestern and central‐western sampling areas, whereas populations in the eastern sampling area (populations 13–32) harbor two haplotypes from the East Clade; however, a mixture of haplotypes from both the West and East Clades was discovered in every population in the northwestern sampling area (Fig. 2C).

Meanwhile, population genetic variations of the nuclear genome are in agreement with the above findings. In a previous survey of *A. tanguticus* using RAPDs, a similar intraspecific divergence was detected, PCA suggested that three populations in the eastern sampling area and six populations in the western sampling area are differentiated from each other relatively well, while one population from the northern‐most part of the eastern sampling area lies between these two groups (Zheng et al. 2008). A similar but slightly different pattern (compared to the pattern of plastid DNA) was also suggested by our ITS‐RFLP data (Fig. 3). Although the gene frequency of ITS‐RFLP type A is significantly higher in the western sampling area and on the southern edge of the eastern sampling area, the genetic composition of most populations in the eastern sampling area is different from those in the western sampling area.

### Intraspecific lineage divergence and glacial habitat fragmentation

The phylogeographic pattern of plastid haplotypes reflects a clear pattern of allopatric intraspecific lineage divergence in the evolutionary history of *A. tanguticus* (Fig. 2C). Either orogenesis events (e.g., the latest extensive uplifts of the QTP) or Quaternary climate changes may have been the cause of intraspecific lineage divergence for plant species on the QTP and in the adjacent area (Wang et al. 2009b, 2009c, 2010; Yang et al. 2012; Yue et al. 2012; Fan et al. 2013; Meng et al. 2015). However, our results suggest that the more likely cause of intraspecific divergence of *A. tanguticus* was isolation of populations that survived in different refugia during Quaternary glacials, rather than geological isolation due to orogenesis events. On one hand, our molecular dating revealed that, based on a mutation rate of plastid DNA for *Anisodus* species of 1.64 × 10^−9^ substitution/site/year, which is moderate among mutation rates for herbaceous plants, the West and East Clades of *A. tanguticus* diverged from each other around the middle Pleistocene (1.69 Mya, 95% HPD: 0.83–2.62 Mya). Such an age estimate is significantly younger than the widely accepted timescale of the latest QTP uplifts (ca. 3.4 Ma), but overlaps with the earliest known Quaternary glacial in the QTP, the Xixiabangma Glacial that occurred ca. 1.6 Mya (see Fig. 2E).

On the other hand, if we consider only high habitat suitability distributions (>0.5), our SDMs for *A. tanguticus* during the LGM under either MIROC (Fig. 4B) or CCSM (Fig. 4C) models suggested in situ survival but severe habitat fragmentation in the species' western sampling area and moderate or obvious eastward migration in its eastern sampling area. Such a trend of habitat fragmentation during the glacial period is strengthened when SDMs with the additional herbarium collecting sites are conducted (Fig. S1B and S1C). In addition, the mountains that lie between the western and eastern sampling areas, as well as within the eastern sampling area, may have acted as physical barriers during the lineage divergence of this species. As shown in Fig. 2C and D, the three strongest dispersal barriers, which fell well in line with the border of the SAMOVA groups, suggested strong isolation between eastern, northwestern, central‐western, and southwestern groups. Note also that plastid haplotypes in the West Clade are confined to the west of barrier I and plastid haplotypes in the East Clade are limited to the north of a combination of barriers I and III. Therefore, it is most likely that the West and East Clades diverged from each other due to habitat fragmentation during the Xixiabangma Glacial, and that they may have survived in different refugia and experienced allopatric divergence. Similar patterns of intraspecific divergence due to Quaternary climate change have also been found in other plant species in the QTP and adjacent regions, such as *Aconitum gymnandrum* (Wang et al. 2009b), *Sophora davidii* (Fan et al. 2013), and *Oxyria sinensis* (Meng et al. 2015).

### Interglacial retreat and subsequent recolonization of the eastern sampling area

Subsequently, we found that distribution range contraction during the LIG in the eastern sampling area of *A. tanguticus* is an important driver for the relatively low level of genetic variation in this region (Figs. 2C, 3); nevertheless, the LIG delivered relatively lower impact on the distribution dynamics of the western populations. As shown in Fig. 4D, our SDMs during the LIG showed a significantly narrower distribution in comparison with the present‐day model. First, as noted above, the highly likely distributions (habitat suitability > 0.5) of *A. tanguticus* are mainly located on the southeastern edge of the QTP, although a potential refugium in the northern‐most distribution range cannot be entirely eliminated (Fig. 4D). Subsequently, populations in the central part of the eastern sampling area probably experienced interglacial retreat (most likely southward) or extinction and subsequent recolonizations, as locations of 14 of 20 populations fell out of the possible distribution (habitat suitability < 0.15) in the LIG model. Note that such a contraction of distribution range is more evident when all herbarium samples are integrated into the SDMs (Fig. S1D). Finally, the interglacial history of populations in the western sampling area is more or less ambiguous, because locations of all of these populations were predicted to be within the possible distribution (0.5 > habitat suitability > 0.15). The range contraction in our LIG models is in agreement but more significant than these of other plants occur in similar area in the QTP (Li et al. 2013; Sun et al. 2015).

The distribution patterns of genetic variation corroborate the SDM models. As shown in Figs. 2C and 3, relatively poor genetic variation in these populations (populations 16–17, 19–30), whose locations were predicted as uncolonized in the LIG model, is consistent with a scenario where these populations experienced recolonization after interglacial retreat/extinction. Due to founder effects, recolonized populations usually possess lower genetic variation (e.g., Petit et al. 2003; Stewart et al. 2010); as shown in Fig. 2C, only a single plastid haplotype was detected in 12 of 14 populations (the locations of which were predicted as not supporting the species during the LIG); there is also a decrease in the number of nuclear ITS‐RFLP types in the central and northern parts of the eastern sampling area (Fig. 3). In contrast, populations in the western sampling area probably experienced in situ survival through the glacial/interglacial maximum and subsequent local expansion. This is in agreement with the strong phylogeographic structure in the western populations (Fig. 2C), which suggests that range expansions were limited to the local scale. The geographic distribution pattern of nuclear ITS‐RFLP types (Fig. 3) is also in agreement with in situ survival and local expansion in the western sampling area.

### Local admixing between plastid lineages in climate refugia

Furthermore, multiple chains of evidence suggest that high haplotype diversity in the northwestern sampling area is a consequence of local lineage admixing and subsequent survival in climate refugia during the Quaternary. Considering that plastid haplotypes from both the West and East Clades were detected in every population in the northwestern sampling area whereas only plastid haplotypes from one clade were detected in other sampling areas, there are probably unidirectional dispersal barriers (in other words there is asymmetric gene flow) between this sampling area and the other sampling areas. As shown in Fig. 2C and D, migrations of plastid haplotypes across barrier I may have occurred only from east to west, whereas migrations across barrier III may have solely occurred from south to north. Notably, the population genetic survey based on RAPD markers suggested that three populations in the northwestern sampling area and three populations in the eastern sampling area are different from each other, but populations in the north‐ and central‐western sampling areas are closer to each other than to populations from the eastern sampling area; our ITS‐RFLP results suggest a similar pattern. Therefore, migrations or gene flow across barrier III was more frequent than across barrier I, facilitating homogenization of biparentally inherited nuclear genomes (via both seed flow and pollen flow) among populations within the western sampling area. Nevertheless, plastid genomes are maternally inherited, their dispersal relies solely on seeds; compared to gene flow via both seeds and pollen, gene flow via seeds may be less effective; therefore, plastid genomes are more prone to interspecific or interlineage gene flow than nuclear genomes (e.g., Petit and Excoffier 2009; Zhou et al. 2010; Du et al. 2011). This explains the divergence of nuclear genome (RAPDs, ITS‐RFLPs) between the eastern and western sampling areas but asymmetric migration of the East Clade plastid haplotypes (most likely from the eastern range) into the western sampling area.

At the same time, it is highly likely that most populations in the northwestern sampling area (especially population 1) survived in situ through the LGM and LIG in climate refugia. An important indicator of climate refugia is higher than average haplotype diversity and the presence of private haplotypes (e.g., Petit et al. 2003; Hewitt 2004; Opgenoorth et al. 2010). As shown in Fig. 2C, three or more plastid haplotypes were detected in populations 1, 2, and 4, and two private haplotypes were detected in population 1. Although these populations fell out of the envelope of high habitat suitability (>0.5) in both SDMs during the LGM and LIG, all of their locations were predicted as being within the potential distribution (>0.15) during the LIG (Fig. 4D), and locations of populations 1, 2, and 4 were predicted as within the potential distribution (>0.15) in both LGM models (Fig. 4B and C). However, due to the adaptive capacity of a species, its ecological niche breadth under climate extremes, such as during a glacial maximum (e.g., the LGM) or an interglacial (e.g., the LIG), may be wider than the current realized niche width (e.g., Catullo et al. 2015). In this study, our SDMs only take into consideration the realized niche, if adaptive capacity of *A. tanguticus* is to be integrated into SDMs, the locations of populations in the northwestern sampling area would be covered by the high habitat suitability envelope in both the LGM and LIG models. Under these circumstances, both genetic data and ecological niche support the presence of climate refugia in the northwestern sampling area.

### Reproductive attributes and the distribution pattern of genetic variation

The reproductive attributes of *A. tanguticus* may also have contributed to its distribution pattern with respect to genetic variation (Zheng et al. 2008). Although this species is mainly outcrossing and self‐incompatible in most cases, it has been observed that selfing and self‐compatibility occurs in populations where outcrossing pollinators are rare in high‐altitude populations or where pollinators facilitate selfing in low altitude populations (Duan et al. 2007). Note that *A. tanguticus* is octoploid (Tu et al. 2005), and polyploidy usually results in decreased self‐incompatibility (and hence, increased self‐compatibility) (Mable 2004) and increased tolerance to inbreeding depression (Husband and Schemske 1997). Therefore, selfing may play an important role in high‐altitude populations and also probably in many populations during Quaternary glacial periods, when outcrossing pollinators were scarce. Such selfing in the breeding strategy may be partially responsible for the high level of genetic differentiation between populations of this species, as detected by RADP markers (Zheng et al. 2008), and the commonly seen heterozygosity of ITS‐RFLP (Fig. 3) and low level of within‐population diversity of cpDNA (Fig. 2) found here. This is because selfing leads to a reduction in both gene flow between different populations and between individuals in the same population (Freeland et al. 2011).

### Conservation and management in the future

*Anisodus tangticus* has a long history in Tibetan folk medicine. It contains high levels of hyoscyamine and scopolamine, two tropane alkaloids that can be used to act on the parasympathetic nervous system as anticholinergic agents to relieve pain (Yang 1991; Zhang and Wang 2002). However, in recent decades, wild populations of the species have experienced drastic decline due to over‐harvesting to extract tropane alkaloids (Yang 1991; Zhang and Wang 2002). To preserve this endemic and threatened species, collection for chemical extraction should be diverted from wild plants to commercially cultivated plants. Although, ideally, any conservation program should involve all known populations, in practice, priority is usually given to populations that harbor higher genetic diversity, higher heterozygosity, and unique genetic components (e.g., Freeland et al. 2011). Following our genetic surveys based on plastid markers, we make three proposals that will facilitate conservation and management of this species. First, the significant phylogeographic structure in *A. tanguticus* (*N*_ST_ = 0.823 ± 0.066, *G*_ST_ = 0.802 ± 0.058; *N*_ST_ > *G*_ST_, 0.01 < *P *<* *0.05), indicates that closely related haplotypes tend to be distributed within the same area; meanwhile, SAMOVA suggests that most genetic variation exists among four groups of populations (*F*_CT_ = 0.834). Therefore, conservation and management programs should be undertaken in each of these four areas (Fig. 2C). Subsequently, western populations (13–32) expanded over a range of latitudinal gradients, and a combination of plastid and nuclear allele frequencies could have led to four genetically differentiated subgroups (populations 14–16, 20; populations 18–19, populations 21–23; populations 25–27; populations 28–32). At least some representative populations from each subgroup should be preserved. Furthermore, the average within‐population diversity *H*_S_ (0.131 ± 0.0426) is much lower than the total genetic diversity *H*_T_ (0.661 ± 0.0643) across all populations, indicating that overall within population plastid diversity is low. However, many populations possess a higher level of haplotype richness, including populations 1–4, 6, 8, 23, 25; thus, these populations should be allocated preservation priority. In addition to these, our genetic survey will also provide useful genetic background when developing commercial cultivars.

In addition to the above suggestions based on genetic diversity, SDMs are also helpful when formulating conservation and management strategies, as they predict potential changes of geographic distribution under future climate change scenarios (e.g., Fordham et al. 2014; Costion et al. 2015; Gotelli and Stanton‐Geddes 2015). In agreement with previous field observations and SDM, which suggested that a warming climate will lead to migration of mountain species toward higher altitudes (e.g., Grabherr et al. 1994; Gottfried et al. 1999; Pauli et al. 2007) and temperate species toward higher latitudes (e.g., Parmesan et al. 1999; Malcolm et al. 2002; Hickling et al. 2006), our SDMs based on the field survey dataset suggested that in both global warming models (2050‐rcp45 and 2070‐rcp85; Fig. 5B, and C), populations that are in areas of low habitat suitability (<0.5) usually occur at lower altitudes or lower latitudes. SDMs based on herbarium samples and the field survey dataset suggest a similar pattern (Fig. S2). In other words, populations from lower altitude or lower latitude areas may face a higher extinction risk than the others, if we consider that populations in areas of low habitat suitability (<0.5) face a higher extinction risk and vice versa. Under a moderate global warming scenario (Fig. 5B), fringe populations such as 5–8, 10, 11, 14, 15, 16, 20, and 29–32 may face a higher extinction risk; strikingly, under an extreme global warming scenario (Fig. 5C), 26 of 32 sampled populations will face a high extinction risk (populations 4–17, 19–26, and 29–32). Doubtless, populations that face a higher extinction risk under global warming scenarios should be of priority for conservation and management of this medicinal plant. In situ and ex situ conservation measures for populations 5–8, 10, 11, 14, 15, 16, 20, and 29–32 should be seriously considered. From another perspective, areas around the convergence of Qinghai, Sichuan, and Gansu provinces (i.e., southeastern Qinghai, northern‐most Sichuan and southwestern Gansu; areas containing populations 17, 23, 27, and 29) all possess high habitat suitability (>0.5), even under the extreme global warming scenario (Fig. 5C); therefore, they should be listed as the core area for the cultivation and conservation of this species.

## Conclusions

In the present study, focusing on a QTP endemic octoploid herbaceous species *A. tanguticus*, we reconstructed its Quaternary evolutionary history based on both phylogeographic pattern and SDMs. This species may have undergone intraspecific divergence during the early Quaternary, which might have been caused by survival in different refugia during the earliest known glacial in the QTP, rather than geological isolation due to the latest extensive QTP uplifts. Its two lineages are distributed mainly in the eastern sampling area and solely in the western sampling area, respectively, but were mixed with each other in the northwestern sampling area. Only one plastid haplotype was detected in most populations in the eastern sampling area, but five of six haplotypes were found in the northwestern sampling area. SDM models for the present, the LGM and the LIG, suggest that the interglacial period may have had a stronger effect than the glacial periods on the recent evolutionary history of this species. While LGM models suggest eastward contraction in the eastern sampling area and severe habitat fragmentation in the western sampling area, the LIG model predicted that this species probably experienced significant interglacial retreat and subsequent recolonization, especially in the eastern sampling area. The geographic distributions of plastid haplotypes and nuclear ITS‐RFLP types corroborate the LIG model, revealing that most eastern populations may have experienced postinterglacial recolonization, at least post‐LIG recolonization. At the same time, a combination of an asymmetric migration barrier (barrier I in Fig. 2D) and in situ survival in climate refugia during the LGM and LIG is the most likely cause for high haplotype diversity and lineage admixing in the northwestern sampling area.

In summary, SDMs are a very useful approach to complement and improve the testing of phylogeographic hypotheses based on population genetic approaches (e.g., Alvarado‐Serrano and Knowles 2014; Gavin et al. 2014), especially in topographically complex areas (e.g., Schorr et al. 2012; Li et al. 2013; Bystriakova et al. 2014). We consider that integration of SDMs with traditional population genetic approaches will significantly improve the robustness of hypotheses proposed with respect to the Quaternary evolutionary history of QTP endemics.

## Conflict of Interest

None declared.

## Supporting information


**Figure S1.** Potential distribution of *A. tanguticus* during the present‐day, the LGM and the LIG based on presence locations according to both herbarium samples and field survey in this study.
**Figure S2.** Potential distribution of *A*. *tanguticus* under future global warming scenarios based on presence locations according to both herbarium samples and field survey in this study.
