# Supplementary material for: The Quaternary evolutionary history, potential distribution dynamics, and conservation implications for a Qinghai–Tibet Plateau endemic herbaceous perennial, Anisodus tanguticus (Solanaceae)
Source: Ecol Evol. 2016 Feb 24;6(7):1977–95. doi: 10.1002/ece3.2019 (PMC4831433; doi:10.1002/ece3.2019)

## *Ecology and Evolution* Supporting Information

Article title: The Quaternary evolutionary history, potential distribution dynamics and conservation implications for a Qinghai-Tibet Plateau endemic herbaceous perennial, *Anisodus tanguticus* (Solanaceae)

Authors: Dong-Shi Wan, Jian-Ju Feng, De-Chun Jiang, Kang-Shan Mao, Yuan-Wen Duan, Georg Miehe, Lars Opgenoorth

The following Supporting Information is available for this article:

Figure S1. Potential distribution of *A. tanguticus* during the present-day, the LGM and the LIG based on presence locations according to both herbarium samples and field survey in this study. Predicted distributions are shown for (a) the present-day model, (b) the LGM-MIROC model, (c) the LGM-CCSM model and (d) the LIG model. White squares indicate presence locations according to our field survey, white pies indicate presence locations based on herbarium samples, and white circles (with a black dot in the center) indicate the main cities.


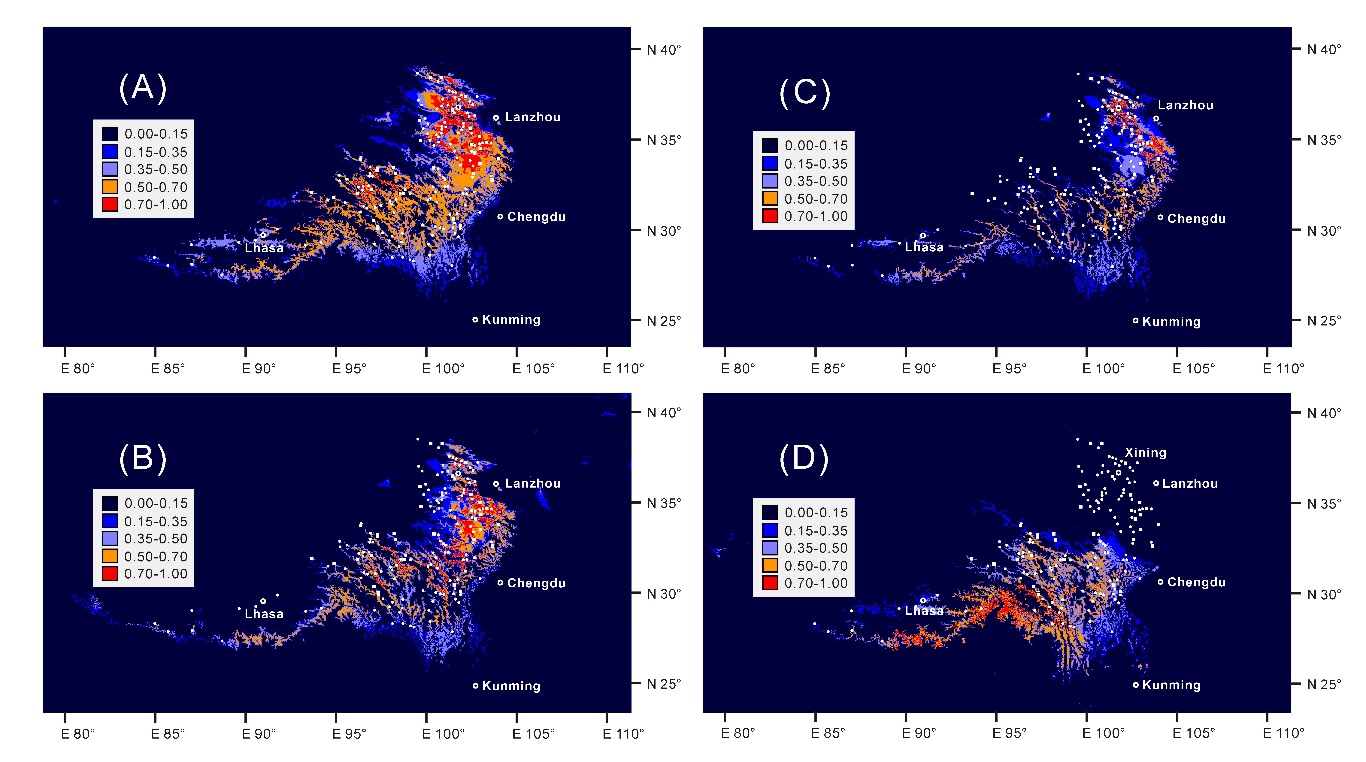


Fig. S2. Potential distribution of *A*. *tanguticus* under future global warming scenarios based on presence according to both herbarium samples and field survey in this study. White squares indicate presence locations according to our field survey, white pies indicate presence locations based on herbarium samples, and white circles (with a black dot in the center) indicate the main cities.


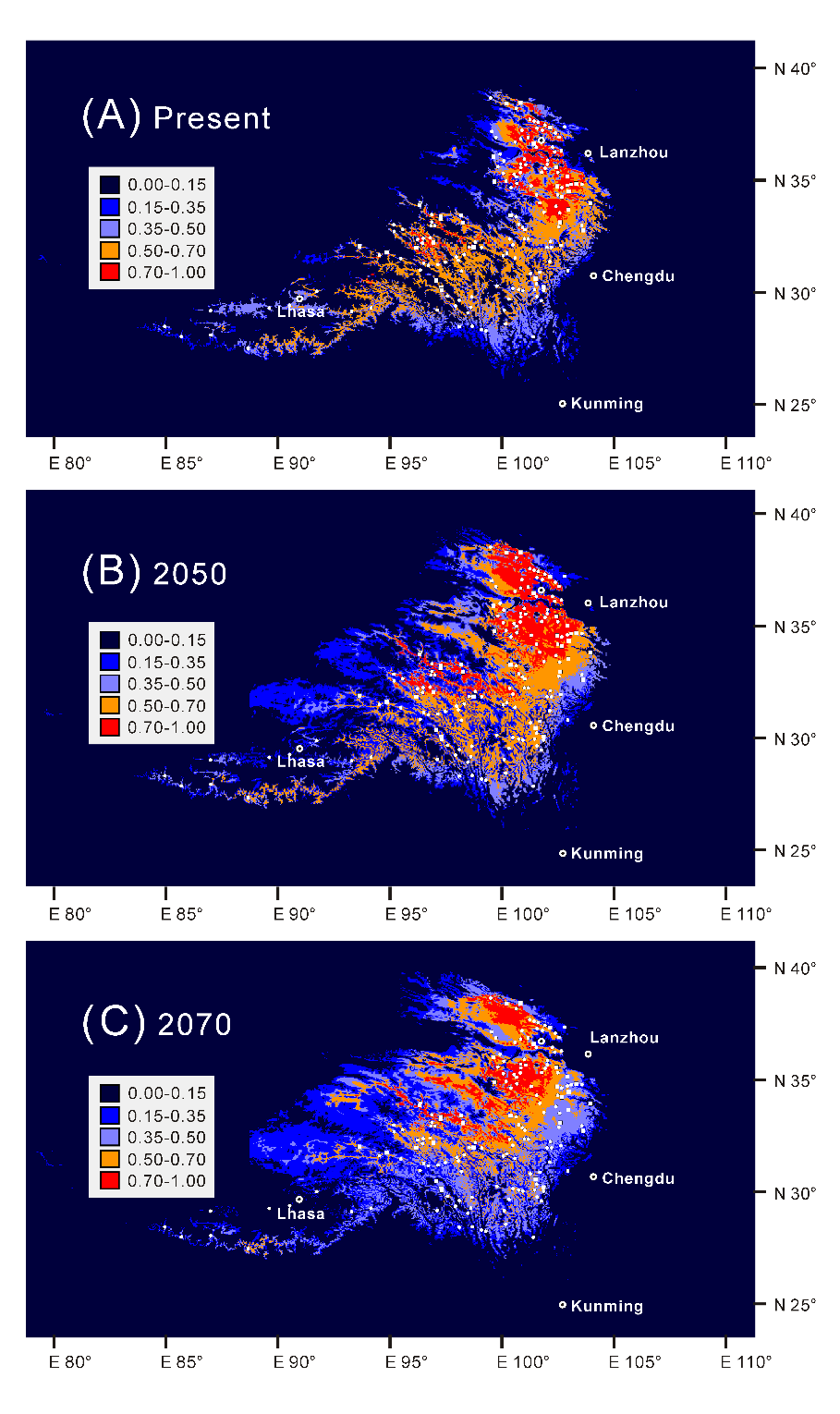

Supplement: Supplementary file 1 — Figure S1. Potential distribution of A. tanguticus during the present‐day, the LGM and the LIG based on presence locations according to both herbarium samples and field survey in this study. Figure S2. Potential distribution of A. tanguticus under future global warming scenarios based on presence locations according to both herbarium samples and field survey in this study. [file ECE3-6-1977-s001.docx]
